# Genome-wide RNA-seq analysis indicates that the DAG1 transcription factor promotes hypocotyl elongation acting on ABA, ethylene and auxin signaling

**DOI:** 10.1038/s41598-018-34256-3

**Published:** 2018-10-26

**Authors:** Riccardo Lorrai, Francesco Gandolfi, Alessandra Boccaccini, Veronica Ruta, Marco Possenti, Anna Tramontano, Paolo Costantino, Rosalba Lepore, Paola Vittorioso

**Affiliations:** 1 Department of Biology and Biotechnology, Sapienza University of Rome, Laboratory affiliated to Istituto Pasteur Italia – Fondazione Cenci Bolognetti, Rome, 00185 Italy; 2 Department of Physics, Sapienza University of Rome, Laboratory affiliated to Istituto Pasteur Italia – Fondazione Cenci Bolognetti, Rome, 00185 Italy; 3 Research Centre for Genomics and Bioinformatics, Council for Agricultural Research and Economics (CREA), Rome, 00178 Italy; 4 Present Address: CIBIO (Centre for Integrative Biology), Universita’ di Trento, 38123 Povo, (TN) Italy; 5 Present Address: Center for Integrative Genomics, Faculty of Biology and Medicine, University of Lausanne, CH-1015 Lausanne, Switzerland; 6 Present Address: SIB Swiss Institute of Bioinformatics, Biozentrum, University of Basel, CH-4056 Basel, Switzerland

## Abstract

Hypocotyl elongation is influenced by light and hormones, but the molecular mechanisms underlying this process are not yet fully elucidated. We had previously suggested that the Arabidopsis DOF transcription factor DAG1 may be a negative component of the mechanism of light-mediated inhibition of hypocotyl elongation, as light-grown *dag1* knock-out mutant seedlings show significant shorter hypocotyls than the wild type. By using high-throughput RNA-seq, we compared the transcriptome profile of *dag1* and wild type hypocotyls and seedlings. We identified more than 250 genes differentially expressed in *dag1* hypocotyls, and their analysis suggests that DAG1 is involved in the promotion of hypocotyl elongation through the control of ABA, ethylene and auxin signaling. Consistently, ChIP-qPCR results show that DAG1 directly binds to the promoters of *WRKY18* encoding a transcription factor involved in ABA signaling, of the ethylene- induced gene *ETHYLENE RESPONSE FACTOR* (*ERF2*), and of the *SMALL AUXIN UP RNA 67* (*SAUR67*), an auxin-responding gene encoding a protein promoting hypocotyl cell expansion.

## Introduction

Once germination is completed, the seedling undergoes photomorphogenesis or skotomorphogenesis, depending on the presence or absence of light^1^.

Photomorphogenic development is characterised by open and expanded cotyledons, short hypocotyls and functional chloroplasts, whereas skotomorphogenesis causes long hypocotyls, closed and unexpanded cotyledons with apical hooks, and undifferentiated chloroplasts (etioplasts).

Hypocotyl elongation is influenced by both environmental (primarily light and gravity) and hormonal cues, and it has been extensively studied as a model for cell elongation. Among phytohormones, auxin plays a pivotal role in promoting cell elongation, and its effect is mediated by the TRANSPORT INHIBITOR RESPONSE1 (TIR1)/AUXIN SIGNALING F-BOX (AFB)-Aux/IAA nuclear auxin receptor and the activation of *SMALL AUXIN UP RNA* (*SAUR*) genes^2,3^. The role of ethylene in hypocotyl development is strictly dependent on light conditions; indeed, in the dark ethylene represses, whereas in red light it promotes hypocotyl elongation^4,5^. Ethylene regulates seedling emergence from the soil through the master positive regulator of ethylene signaling ETHYLENE INSENSITIVE3 (EIN3), which is stabilised through COP1-mediated degradation of the F-box proteins EIN3-BINDING F BOX PROTEIN 1 and 2 (EBF1 and 2)^6,7^. EIN3 induces two downstream signaling pathways, respectively mediated by PHYTOCHROME INTERACTING FACTOR3 (PIF3) and ETHYLENE RESPONSE FACTOR 1 (ERF1). Once the seedling perceives the light, ethylene signaling is switched off by phyB-mediated EIN3 degradation^7^.

Abscissic acid (ABA) is known as a growth-inhibitory hormone, although some reports described a stimulatory effect of this hormone in maize, wheat, rice and Arabidopsis^8–10^. Recently, it has been proposed that the stimulatory or inhibitory response to exogenous ABA depend on both doses of, and tissue sensitivity to this hormone^11^. Hayashi and collaborators^12^ have shown that in dark-grown Arabidopsis seedlings ABA reduces the phosphorylation levels and consequently the activity of the plasma membrane H+-ATPase that triggers growth of the hypocotyl.

Although hypocotyl elongation has been extensively described, the molecular mechanisms underlying the hormonal regulation of this process are not yet fully elucidated.

The Arabidopsis Dof transcription factor Dof AFFECTING GERMINATION 1 (DAG1) is a repressor of light-mediated seed germination acting downstream of the master repressor PIF1^13,14^; accordingly, *dag1* knock-out mutant seeds require less GAs and lower red light fluence rates than wild type seeds to germinate^15,16^.

More recently, we demonstrated that DAG1 plays a key role in the control of the developmental switch between seed dormancy and germination^17^, acting on ABA and GA levels to establish (and maintain) seed dormancy and repress germination. DAG1 negatively controls the ABA catabolic gene *CYP707A2* and the GA biosynthetic gene *GA3ox1* through direct binding to their promoters. Consistently, in *dag1* mutant seeds the ABA level is reduced while the GA level is increased compared to the wild type^17^. We had also shown that light-grown *dag1* mutant seedlings have hypocotyls significantly shorter than the wild type, suggesting that DAG1 is a negative regulator in the light-mediated inhibition of hypocotyl elongation^14^.

Here, we investigated the role of DAG1 in the light-mediated inhibition of hypocotyl elongation by analyzing the transcriptome profile of 4 days-old *dag1* and wild type hypocotyls and whole seedlings by means of high-throughput RNA-sequencing.

## Results

### Inactivation of *DAG1* reduces hypocotyl cell elongation

**Figure 1:**
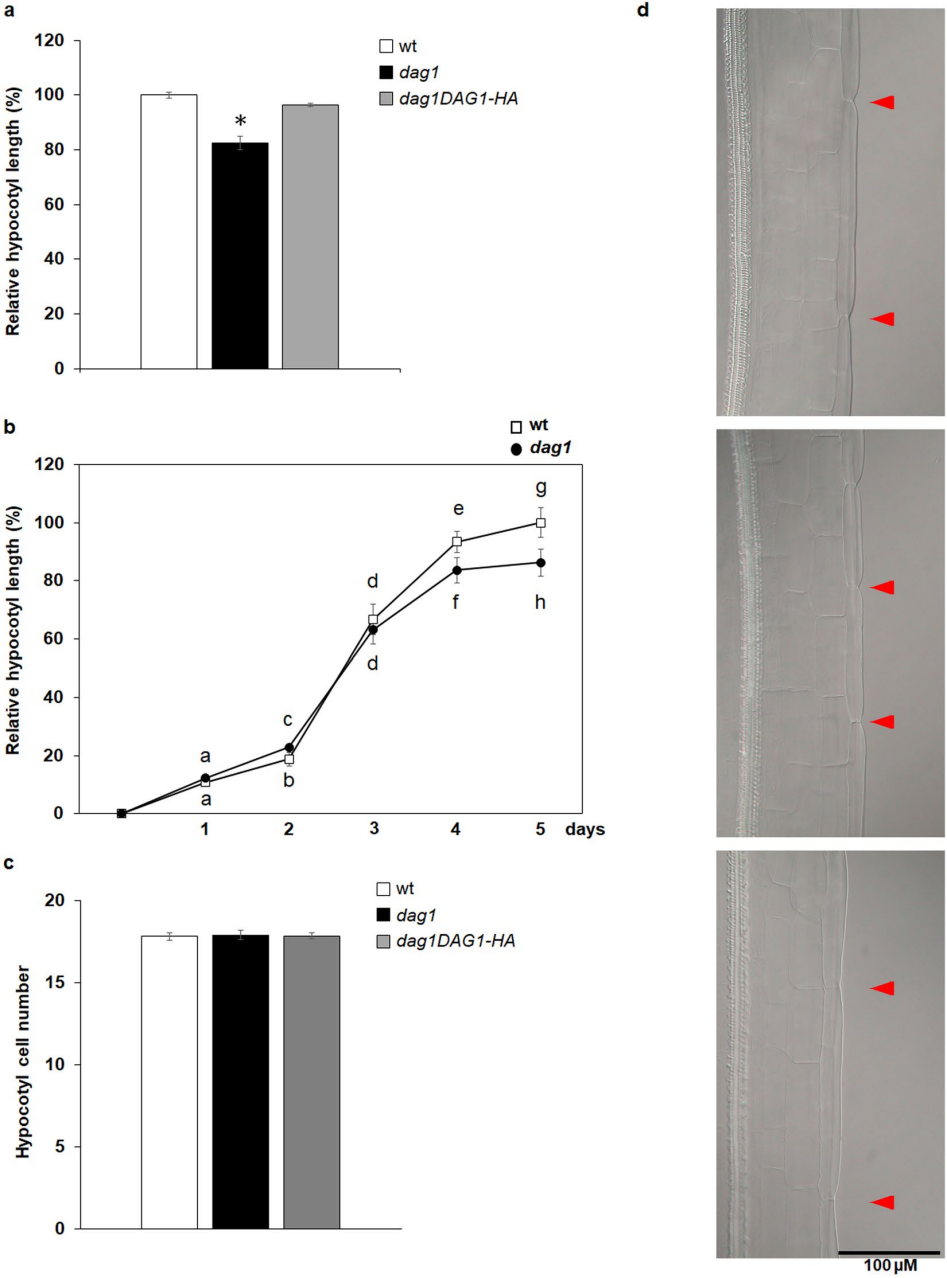
*DAG1* inactivation affects hypocotyl cell expansion. (**a**) Hypocotyl length of *dag1* (black bar), *dag1DAG1-HA* (grey bars) and wild type (white bar) five days-old seedlings, grown under under continuous monochromatic red light (40 μmolm^−2^s^−1^). (**b**) Hypocotyl growth of *dag1* and wild type seedlings. Hypocotyl length was measured every day up to five days, using IMAGEJ software. Stratified seeds were induced to germinate under white light for 24 h, then grown for 5 days under continuous monochromatic red light (40 μmolm^−2^s^−1^). Three independent biological replicates were performed with SD values (n > 30). Significant differences were determined using two-way ANOVA followed by Tukey post-hoc test; significantly different groups are indicated by the letters. (**c**) Epidermal cell number of *dag1* (black bar), *dag1DAG1-HA* (grey bars) and wild type (white bar) hypocotyls of four days-old seedlings grown on horizontal plates under continuous red light (40 μmolm^−2^s^−1^). For each sample, the number of cells in an epidermal cell file without stomata was counted. The values are the mean of three biological replicates, presented with SD values. Significant differences were analyzed by *t*-test. (**d**) Epidermal cells of wild type, *dag1*, and *dag1DAG1-HA* hypocotyls (top to bottom) of four days-old seedlings. The picture is referred to the third cell of the hypocotyl from the apex. Seedlings were grown as in (**c**).

We have previously shown that *dag1* mutant seedlings grown under continuous red light have significantly shorter hypocotyls compared to wild type^14^. To further corroborate this result we measured hypocotyl length of an Arabidopsis line overexpressing the DAG1-HA chimeric protein in a *dag1* mutant background (*dag1DAG1-HA*)^14^. Five days-old *dag1DAG1-HA* seedlings grown under red light showed hypocotyls of the same length of wild type ones, suggesting that the chimeric protein DAG1-HA is functional and complements the hypocotyl phenotype of the *dag1* mutant (Fig. 1a).

Daily measurements of hypocotyl length for five days under red light revealed that at two days *dag1* hypocotyls were slightly longer than wild type, possibly due to their faster germination rate^15^. At three days, hypocotyl length of mutant and wild type seedlings were comparable; at four and five days *dag1* hypocotyls were significantly shorter than wild type ones (Fig. 1b).

Most of the hypocotyl cells derive from the embryo, and hypocotyl growth is mainly due to longitudinal expansion^18^. To assess whether the *dag1* short-hypocotyl phenotype was due to a reduced number of cells or to decreased cell elongation, the number of hypocotyl epidermal cells was counted in four days-old *dag1, dag1DAG1-HA* and wild type seedlings grown under red light. This analysis revealed that *dag1, dag1DAG1-HA* and wild type hypocotyls do not show a significantly different number of epidermal cells (Fig. 1c). However, while *dag1DAG1-HA* cells are of the same size of wild type ones, *dag1* epidermal cells are significantly shorter (Fig. 1d).

### Inactivation of *DAG1* affects several classes of (hormone-related) genes in hypocotyls

To elucidate the role of *DAG1* in the control of hypocotyl growth, we performed RNA-seq analysis of 4 days-old *dag1* and wild type hypocotyls and whole seedlings grown under continuous red light. Three biological replicates of each sample were sequenced using the Illumina Hi-seq platform. For each sample, more then 90% of reads successfully mapped to unique regions of the Arabidopsis genome (TAIR10) (Supplementary Table S1). To evaluate reproducibility among biological replicates, we performed a correlation analysis on normalized gene expression values (CPM, counts per million, see Methods). High positive correlation (Spearman’s correlation coefficient >0.95) was observed between the replicates of each sample (Supplementary Figs S1–S4). Clustering analysis of expression values led to a clear separation of samples according to tissue of origin (hypocotyls vs whole seedlings) as well as to sample condition (*dag1* vs WT) (Supplementary Fig. S5a). However, the latter separation is less apparent in the case of hypocotyls, probably due to the higher gene expression variability observed in *dag1* hypocotyls, as evidenced by Principal Component Analysis (Supplementary Fig. S5b).

**Figure 2:**
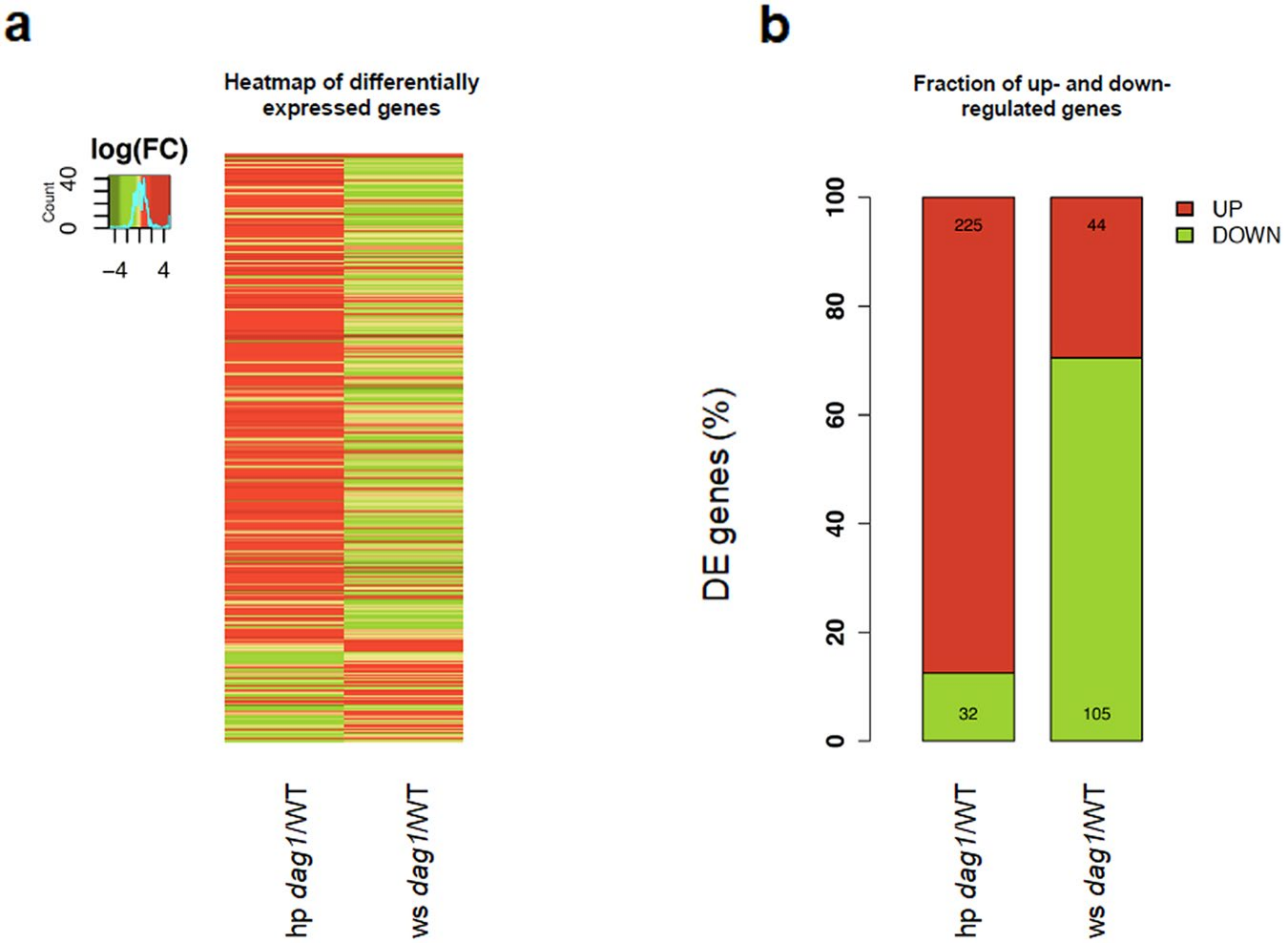
Differentially expressed genes in *dag1* hypocotyls and whole seedlings vs wild type. (**a**) Heatmap of differentially expressed genes. The colour scale indicates higher (red) to lower (green) gene expression levels. Gene expression values are expressed as log2 fold change. (**b**) Fraction of up- and down-regulated genes.

Two comparison groups were constructed and differential expression analysis performed within each group: 1) *dag1* vs wild type hypocotyls (hp *dag1*/WT) and 2) *dag1* vs wild type whole seedlings (ws *dag1*/WT). The overall results of the differential expression analysis are shown in Fig. 2a, where all genes showing a significant expression change (False Discovery Rate, FDR < 0.05) in at least one comparison group are reported, for a total of 388 DE genes (Additional File 1).

The comparison of *dag1* and wild type hypocotyls (hp *dag1*/WT) revealed 257 differentially expressed (DE) genes. Of these, the majority (225) show up-regulated expression in *dag1* (Fig. 2b).

**Figure 3:**
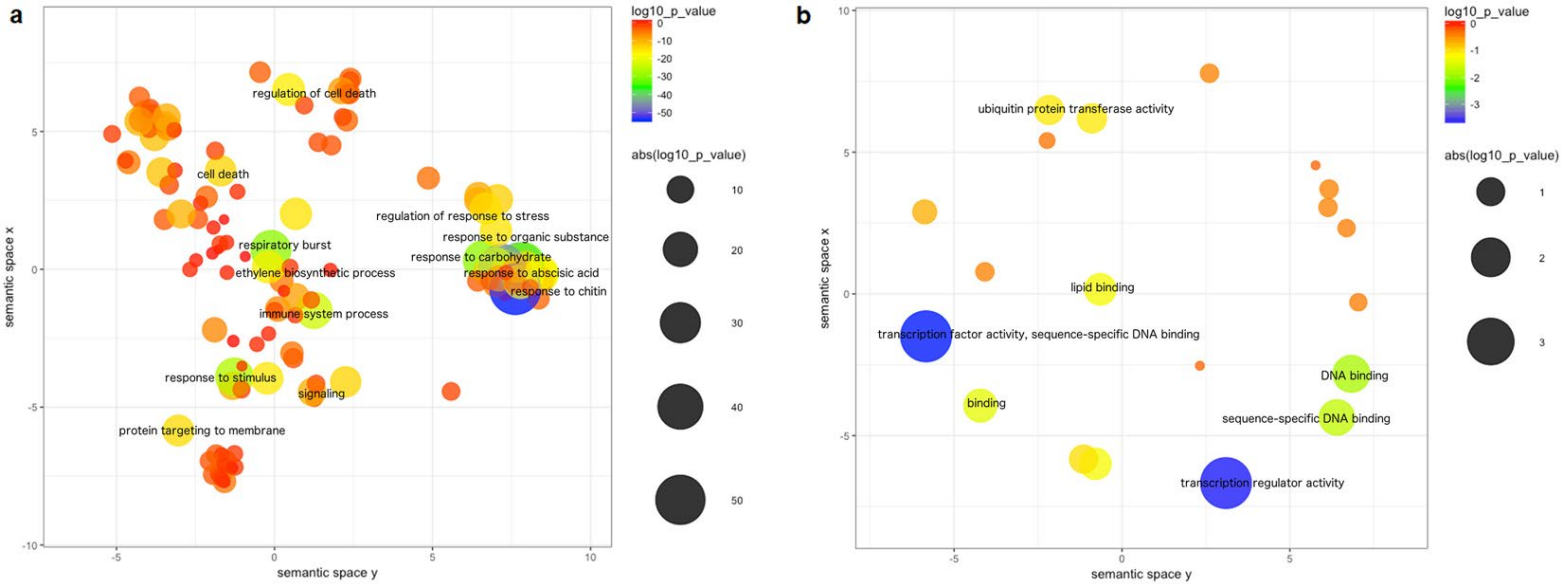
Functional enrichment analysis of up-regulated DE genes in *dag1* vs wild type hypocotyls. (**a**,**b**) Scatterplot view of enriched GO terms for Biological Process (**a**) and Molecular Function categories (**b**) according to REVIGO. Each bubble represents a representative GO term based on semantic similarity clustering. Bubbles are coloured according to p-values and their size indicates the absolute log10 (p-value). Bubbles x and y coordinates are derived by multidimensional scaling of GO term semantic similarity matrices so that more semantically similar GO terms are also closer in the two-dimensional plot.

Results of the functional enrichment analysis of DE up-regulated genes are shown in Fig. 3, where representative enriched Gene Ontology (GO) terms are reported for the Biological Process and Molecular Function categories according to REVIGO. Among the most significantly enriched processes we found different representative terms related to plant response to organic substances (“response to chitin”, “response to carbohydrate”, “response to organic substance”), stimuli (“response to stimulus”, “response to endogenous stimulus”) and immune response (Fig. 3a). A further inspection of these clusters revealed a subgroup of phytohormones-mediated responses, i.e. “response to hormone”, “hormone-mediate signalling pathway”, “response to auxin”, clustering together in the broader category “response to carbohydrate”. Instead, “response to abscisic acid”, “ethylene biosynthetic process” as well as “response to ethylene” form separate clusters and are also significantly enriched (Additional File 2). Notably, ABA, ethylene and auxin are known to be involved in hypocotyl elongation^12,19,20^. Other significantly enriched processes are “respiratory burst” and “immune system process”, possibly suggesting the involvement of DAG1 in the response to environmental stress (Fig. 3a). Finally, as shown in Fig. 3b, enrichment analysis in the Molecular Function category revealed the following significantly enriched GO terms: “transcription factor activity” and “transcription regulator activity” (Fig. 3b; Additional File 2).

The analysis of the down-regulated genes (32 out of 257 DE genes) revealed two enriched functional categories related to hormone response, i.e. “response to hormone” and “response to endogenous stimulus”, however the results are not statistically significant after correction for multiple testing (Additional File 2).

The analysis on whole seedlings (ws *dag1*/WT) identified 149 DE genes, most of them down-regulated in the *dag1* mutant (105 out of 149) (Fig. 2b) (Additional File 1). The functional enrichment analysis on the latter genes revealed few enriched biological processes (FDR<0.05), where the most significantly enriched terms are “response to salicylic acid stimulus”, “defence response” as well as those related to immune response processes, again suggesting a possible involvement of DAG1 in the response to environmental stress (Additional File 2). No significant enrichment was observed in the Molecular Function category, while “extracellular region” resulted to be significantly enriched in the Cellular Component category. Finally, functional enrichment analysis of up-regulated genes revealed significantly enriched biological processes related to plant response to external/extracellular stimuli, nutrient levels, starvation and chemical homeostasis.

In summary, the results of our differential expression analysis between *dag1* and wild type are consistent with the role of DAG1 as a transcription factor and suggest its involvement in the control of hormone-related genes in hypocotyls as well as in response to stress in the whole seedling. Moreover, a differential effect is observed on gene expression levels between hypocotyls and whole seedlings, resulting in a more prominent alteration of biological processes in hypocotyls.

#### *Hormone-related genes*

The RNA-seq data suggest that DAG1 is involved in the control of ABA, ethylene and auxin-related genes in hypocotyls. We validated the expression of a number of these hormone-related genes by RT-qPCR on *dag1* and wild type hypocotyls.

Of the ABA-related genes, we analysed the expression of *RAB GTPASE HOMOLOG B18* (*RAB18*), a stress-responsive gene involved in ABA and drought response^21,22^, *ABA REPRESSOR1* (*ABR1*) encoding an APETALA2 (AP2) domain transcription factor known as a repressor of ABA^23^ and the ABA-responsive WRKY40 transcription factor encoding gene^24^. Among the genes enriching the “response to ethylene” biological process, we validated the expression of five ETHYLENE RESPONSE FACTORS (ERF)-encoding genes (*ERF2*, *ERF5*, *ERF11*, *ERF105*, *ERF109*, Additional File 2). This large family of transcription factors includes proteins with very diverse functions, involved in ethylene, ABA and gibberellins signaling^25,26^.

Four *SAUR* genes - auxin-induced genes highly expressed in hypocotyls^2^ where they promote hypocotyl elongation - namely *SAUR50*, *SAUR63*, *SAUR65*, and *SAUR67* were among the DE genes belonging to the “response to auxin stimulus” enriched process (Additional File 2).

**Figure 4:**
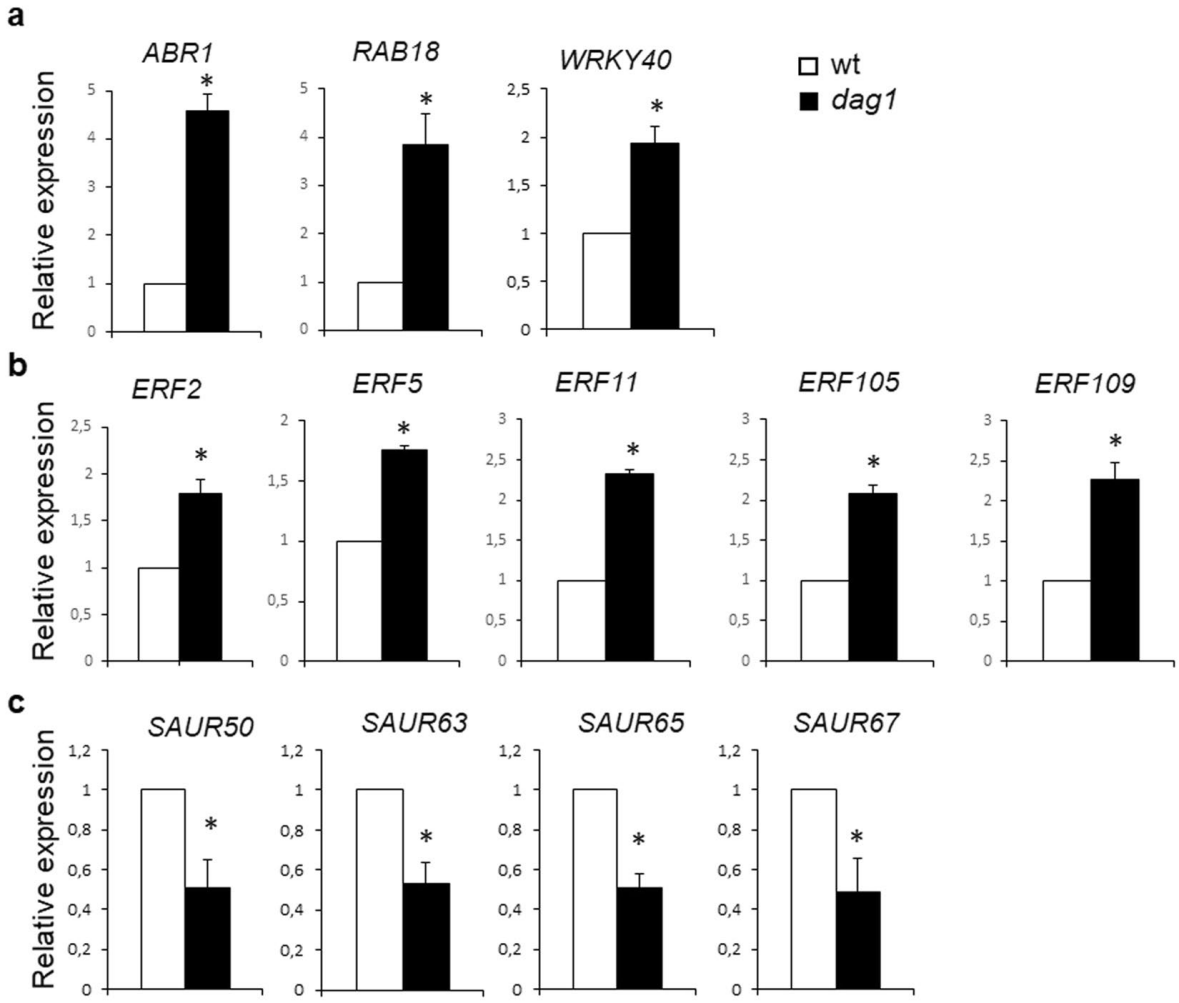
*DAG1* inactivation affects expression of ABA, ethylene and IAA-related genes. (**a**–**c**) Relative expression level of: *RAB18*, *ABR1* and *WRKY40* (**a**) *ERF2*, *ERF5*, *ERF11*, *ERF105*, and *ERF109* (**b**) *SAUR50*, *SAUR63*, and *SAUR65* and *SAUR67* (**c**) in *dag1* and wild type hypocotyls from four days-old seedlings grown under continuous monochromatic red light (40 μmolm^−2^s^−1^). RT-qPCR assays were performed with 1 μl of the diluted cDNA, along with the specific primers, listed in Table S3. Relative expression levels were normalized with *UBQ10* (At4g05320) reference gene. The values of relative expression levels are means of three biological replicates, presented with SD values. Significant differences were analyzed by *t*-test (*P ≤ 0,05).

The results of this RT-qPCR analysis confirmed the RNA-seq data: the transcript levels of the ABA- and ethylene-related genes were higher and those of the *SAUR* genes were lower, respectively, in *dag1* than in wild type hypocotyls (Fig. 4a–c), thus supporting the suggestion that the transcription factor DAG1 is involved in the hormonal regulation - specifically ABA, ethylene and auxin - of hypocotyl elongation.

#### *WRKY genes*

**Figure 5:**
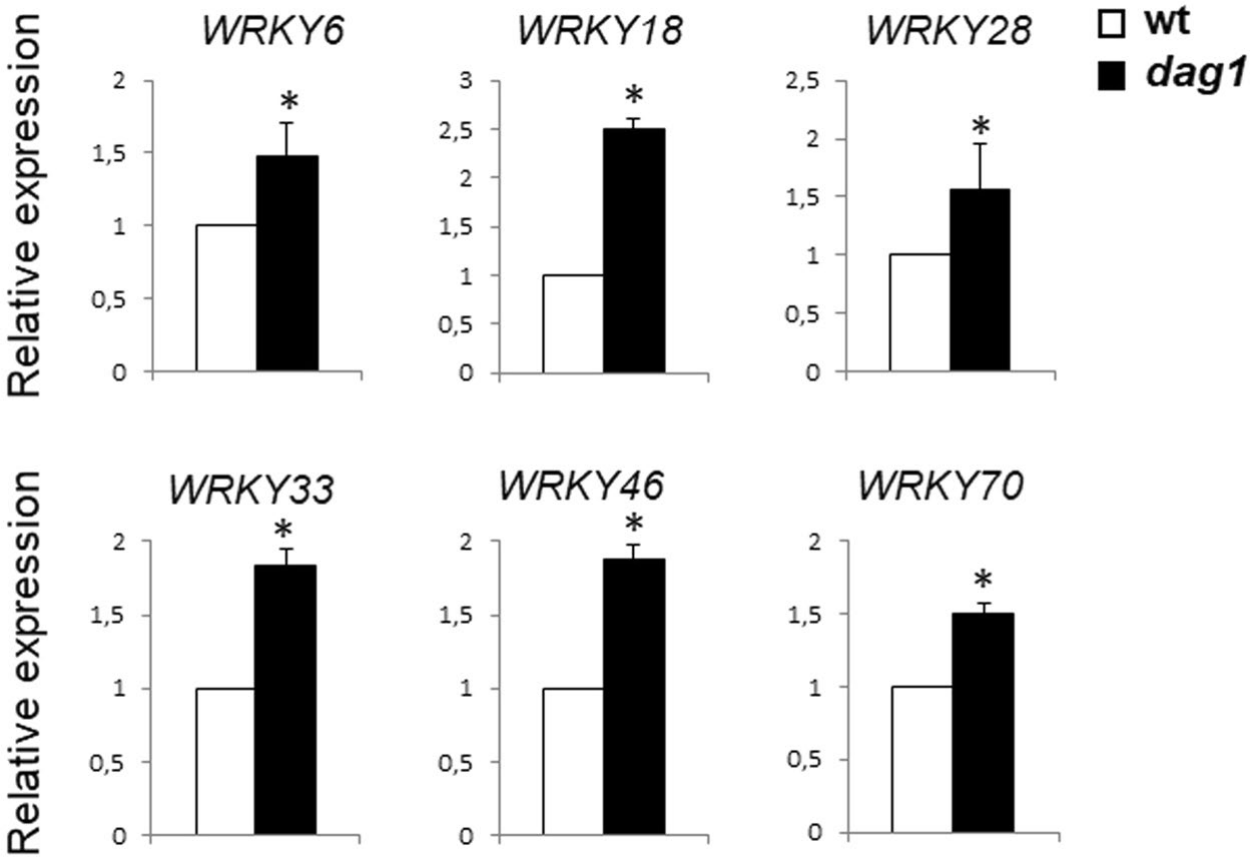
*DAG1* inactivation affects expression of *WRKY* genes in hypocotyls. Relative expression level of *WRKY6*, *WRKY18*, *WRKY28, WRKY33, WRKY46* and *WRKY70* in *dag1* and WT hypocotyls from 4 days-old seedlings grown under monochromatic red light. RT-qPCR assays were performed with 1 μl of the diluted cDNA, along with the specific primers, listed in Supplementary Table S3. Relative expression levels were normalized with *UBQ10* (At4g05320) reference gene. The values of relative expression levels are means of three biological replicates, presented with SD values. Significant differences were analyzed by *t*-test (*P ≤ 0,05).

Among the DE genes in *dag1* hypocotyls, we also found the *WRKY* transcription factor family, encoding key regulators of many plant processes including the responses to abiotic stresses and to ABA, and seed dormancy/germination^27^. Since DAG1 plays a pivotal role in establishing seed dormancy and repressing seed germination by modulating both ABA and GA level, we set to validate these results by analysing the expression of *WRKY6*, *WRKY18*, *WRKY28, WRKY33*, *WRKY46* and *WRKY70* in *dag1* and wild type hypocotyls by RT-qPCR. This analysis confirmed that inactivation of *DAG1* results in a significantly increased expression of the WRKY encoding genes (Fig. 5).

### DAG1 directly binds the promoters of *ERF, SAUR* and *WRKY* genes

DAG1 is known to bind, as all the Dof transcription factors, the CTTT sequence on the promoter of target genes^14,17,28^. Therefore, the validated set of DE genes was analysed for the presence of the CTTT binding site (BS) in their promoter by means of Promomer^29^. Significant matches were found for the *ERF2, SAUR67* and *WRKY18* promoters, suggesting that they could be direct targets of DAG1.

**Figure 6:**
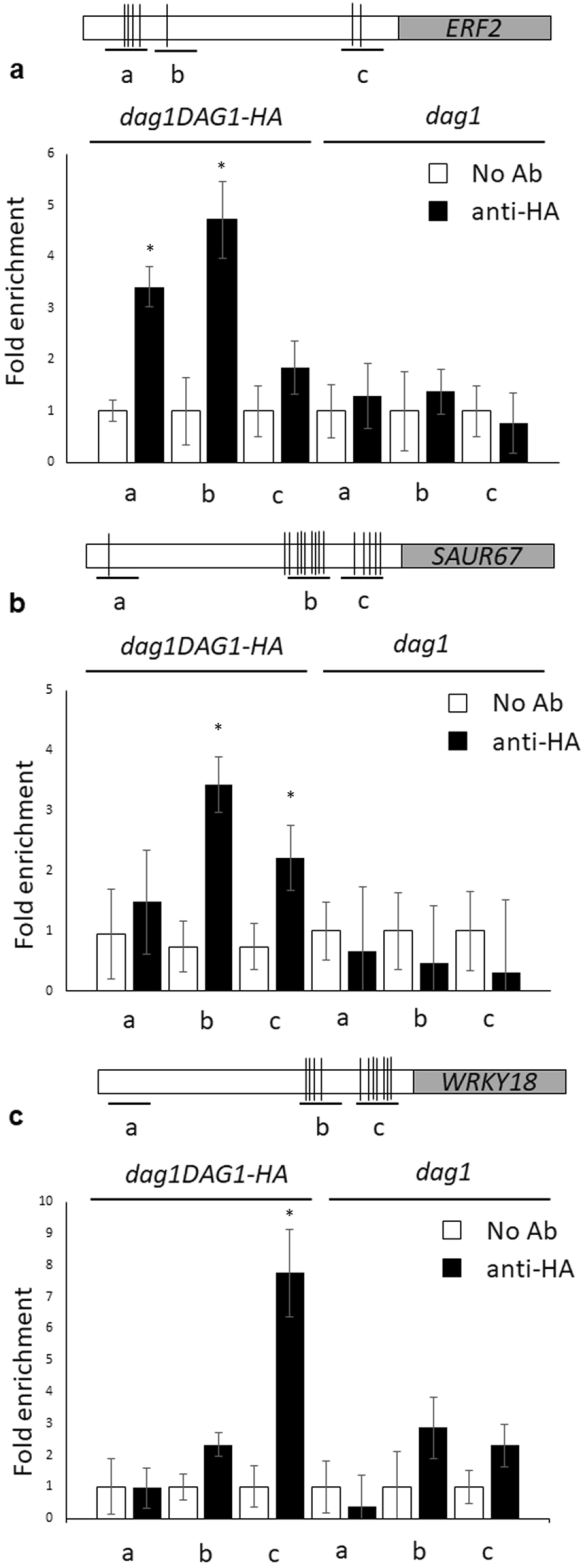
DAG1 directly binds the promoters of *ERF2, SAUR67* and *WRKY18*.(**a**–**c**) Top: graphic representation of the *ERF2* (**a**) *SAUR67* (**b**) and *WRKY18* (**c**) promoters. Underlying thick lines marked by letters (**a**–**c**) are referred to different promoter fragments used for qPCR, containing 4, 1, 2 (*ERF2*), 1, 9, 5 (*SAUR67*) and 0, 4, 7 (*WRKY18*) Dof binding sites respectively. Bottom: chromatin from *dag1DAG1-HA* (left) and from *dag1* (right) seedlings, as a negative control, was immunoprecipitated with anti-HA antibodies, and the amount of DNA was measured by qPCR for the *ERF2* (**a**) *SAUR67* (**b**) and *WRKY18* (**c**) promoter fragments. Similar results were obtained from two independent biological replicates, The values are the average of two biological replicates presented with SD values. Significant fold enrichments were analyzed by t-test (*P ≤ 0,05).

We performed ChIP assays, using *dag1DAG1-HA* plants^14,17,30^. Protein–DNA complexes were precipitated with anti-HA antibodies, or without antibodies as a negative control. As additional negative control, we performed the same assays on untransformed *dag1* seedlings. Three regions of the *ERF2, SAUR67* and *WRKY18* promoters were amplified by qPCR (Fig. 6a–c).

As for *SAUR67* and *WRKY18*, the relative amount of promoter fragments b and c (9, 5 and 4, 7 Dof BS in *SAUR67* and *WRKY18*, respectively) precipitated by DAG1-HA were significantly higher than the negative controls, whereas the enrichment of precipitated promoter fragment a (1 and 0 Dof BS in *SAUR67* and *WRKY18*, respectively) was very low in DAG1-HA and in the negative controls (Fig. 6b,c).

Of the three *ERF2* promoter fragments (with 4, 1 and 2 Dof BS, respectively), amplification of fragments a and b was the most efficient, compared to the negative controls (Fig. 6a). Although only a single Dof site is present in fragment b, it is located within an optimal sequence context - it has been reported that the sequences flanking the Dof BS may influence DNA binding of the Dof proteins^31^.

This analysis indicates that DAG1 directly binds to the *ERF2*, *SAUR67* and *WRKY18* promoters in seedlings.

Upstream regions (500 bp) of the 257 DE genes found in the hp *dag1*/WT comparison group were used as input for the MEME discriminative motif discovery tool^32^ to identify patterns shared among, and/or repeated within these sequences.

Two enriched motifs were identified in some of the promoter sequences, i.e. CACGTG (E-value = 4.6e^−013^) and CTCTCTCT (E-value = 5.5e^−004^) (Supplementary Fig. S6). The former corresponds to a known bHLH-binding motif, i.e. the G-box that is bound by the PIF proteins, a family of transcription factors involved in light-mediated developmental processes^33,34^. Interestingly, *DAG1* has been shown to be positively regulated by PIF1, the master repressor of seed germination^13,14^. PIF1, as well as PIF3, PIF4, PIF5 and PIF7, is also involved in the promotion of hypocotyl elongation^35,36^. Among the genes carrying the G-box motif in their promoters we identified seven *LEA* genes, five *ERF* genes and four *SAUR* genes, which we have previously validated by RT-qPCR. Interestingly, *ERF2* and *SAUR67* show both the PIF and the Dof binding sites (Additional File 3).

### ABA response is altered in *dag1* seedlings

The work described in the previous paragraphs suggests that DAG1 promotes hypocotyl cell elongation and represses the expression of ABA-responsive genes in hypocotyls.

**Figure 7:**
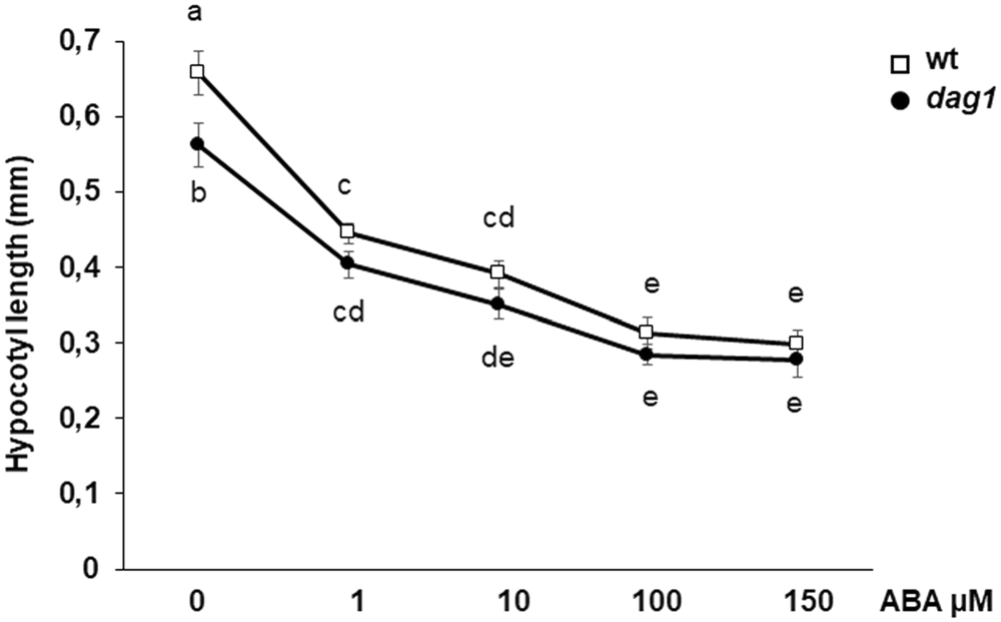
ABA response in *dag1* mutant seedlings. Hypocotyl length of *dag1* and wild type seedlings in the presence of increasing concentration of ABA. Seeds were sown on MS agar with one layer of filter paper 595 (Schleicher & Schull, Dassel, Germany), and 48 h after stratification, seedlings were transferred to plates containing different ABA concentrations (0, 1, 10, 100 and 150 µM). Hypocotyl length was measured after 5 days. Three independent biological replicates were performed, with SD values (n > 30). Significant differences were determined using two-way ANOVA followed by Tukey post-hoc test; significantly different groups are indicated by the letters.

Thus, we wondered whether DAG1 promotes hypocotyl elongation by repressing ABA-mediated inhibition. To substantiate this hypothesis, we measured hypocotyl length in *dag1* and wild type seedling grown under red light in the presence of increasing ABA concentrations (0, 1, 10, 100, 150 µM). As shown in Fig. 7, while at 0 and 1 µM ABA *dag1* hypocotyls are significantly shorter than the wild type, the difference becomes not significant at higher concentrations - thus ABA compensates the lack of DAG1 activity - suggesting that indeed the short-hypocotyl phenotype of *dag1* is amenable to the inhibition of ABA-responsive genes (Figs 7 and S7).

### Analysis of overlapping DE genes between whole seedlings and hypocotyls comparison groups

**Figure 8:**
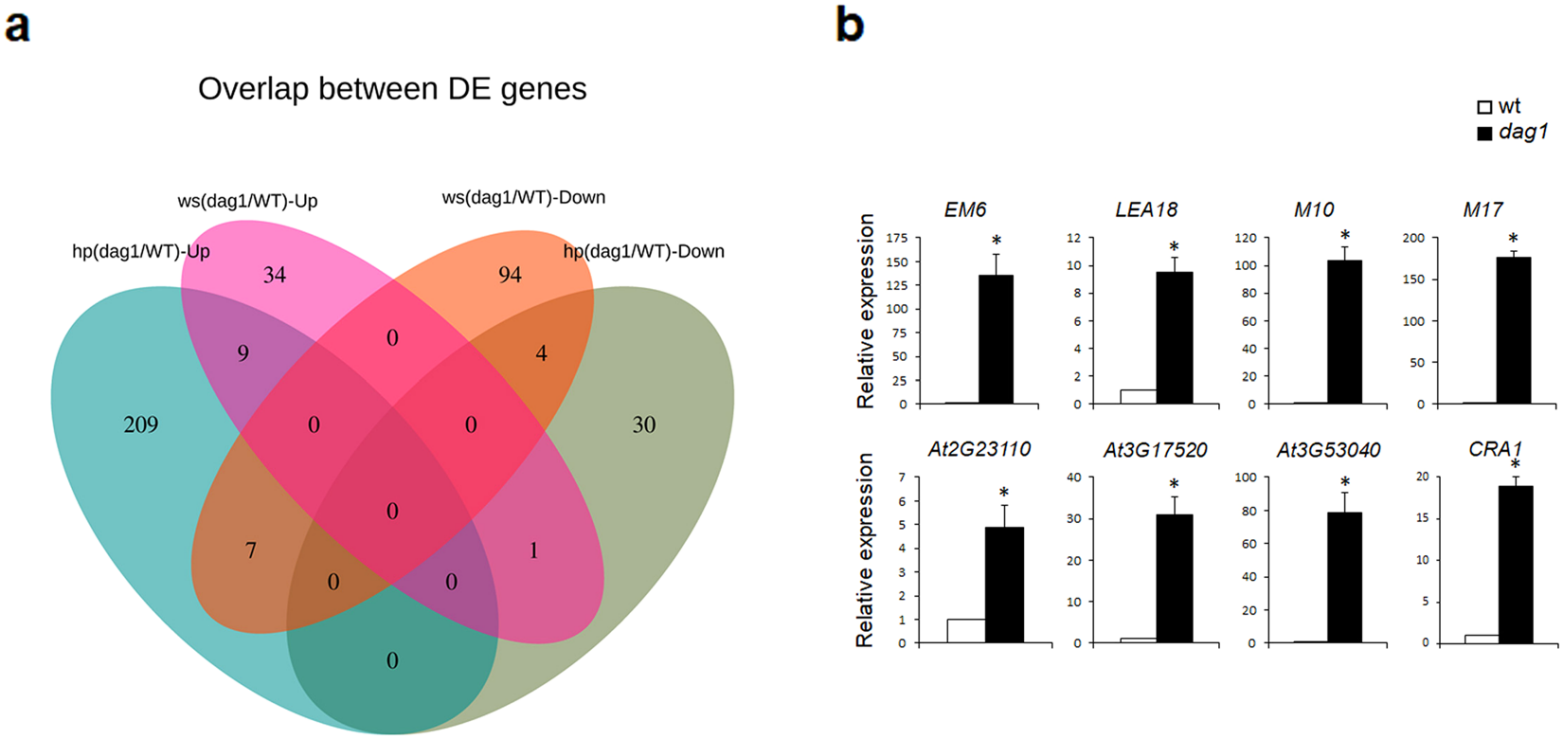
Venn diagram for the up- and down-regulated genes. (**a**) Venn diagram of the hp *dag1*/WT and ws *dag1*/WT up- and down-regulated DE genes. (**b**) Expression analysis of the *M17*, *CRA1, EM6*, *LEA18*, *M10*, At2g23110, At3g17520, and At3g53040 DE genes in *dag1* and wild type hypocotyls under continuous monochromatic red light (40 μmolm^−2^s^−1^). RT-qPCR assays were performed with 1 μl of the diluted cDNA, along with the specific primers, listed in Supplementary Table S3. Relative expression levels were normalized with *UBQ10* (At4g05320) reference gene. The values of relative expression levels are means of three biological replicates, presented with SD values. Significant differences were analyzed by *t*-test (*P ≤ 0,05).

Our transcriptome analysis revealed an overlapping set of differentially expressed genes showing opposite expression change between the two comparison groups (Fig. 8a).

More specifically, the gene encoding the *microRNA167D* (*miR167D*), is down-regulated in hp *dag1*/WT and up-regulated in the ws *dag1*/WT group; the reads corresponding to *miR167D* map on a region corresponding to the stem–loop structure containing miRNA and complementary miRNA sequences (pre-miRNA) (Supplementary Fig. S8), suggesting that lack of DAG1 affects *miR167D* at the transcriptional and/or processing level.

Viceversa, 7 DE genes are up-regulated in hp *dag1*/WT and down-regulated in ws *dag1*/WT. The two seed-specific genes *Late Embryogenesis Abundant Protein* (*LEA*) gene *M17* and *Cruciferin 1* (*CRA1)* were among these genes (Fig. 8a). By means of RT-qPCR on *dag1* and wild type hypocotyls, we validated the expression of these genes.

Since DAG1 has been previously shown to play a pivotal role during seed development^17,37^, we validated also the expression of other six *LEA* genes up-regulated in the hp *dag1*/WT group (Additional File 1; Supplementary Table S2). The expression analysis corroborated the RNA-seq data as in *dag1* mutant hypocotyls the expression of these genes was sharply increased compared to wild type, thus suggesting that DAG1 is required to repress these seed-specific genes during hypocotyl development (Fig. 8b).

## Discussion

We have previously demonstrated that DAG1 is involved in the repression of light-mediated inhibition of hypocotyl elongation^14^. Here, we show that DAG1 promotes hypocotyl cell expansion; based on the transcriptome analysis of *dag1* hypocotyls, we suggest it does so through the control of ABA, ethylene and auxin signaling.

Indeed, the functional enrichment analysis of the DE up-regulated genes of the *dag1*/WT hypocotyl comparison, revealed that “response to abscisic acid”, “response to ethylene” and “response to auxin” are among the significantly enriched processes.

ABA, a growth-limiting hormone, has been shown to suppress hypocotyl elongation in etiolated squash hypocotyl segments^38^, as well as in etiolated Arabidopsis seedlings^12^. In addition, it has been recently demonstrated that ABA-responsive genes are repressed in shade avoidance-driven hypocotyl elongation^39^.

Among the up-regulated genes of the *dag1*/WT hypocotyl comparison group falling in the “response to abscisic acid” biological process and whose expression we validated by RT-qPCR were *RAB18* and *ABR1*, encoding a GTPase and a transcription factor respectively, involved in ABA signaling^21–23^. Our results suggest that DAG1 is required to repress the expression of these genes in hypocotyls, consistent with the *dag1* short-hypocotyl phenotype. In addition, our RNA-seq analysis identified seven WRKY transcription factors-encoding genes that are up-regulated in *dag1* hypocotyls. The WRKY transcription factors are known as key components of ABA signaling: WRKY18, and the two closely related WRKY60 and WRKY40 proteins, have been shown to cooperate in plant response to biotic stress with both overlapping and distinct functions^40,41^. As for the response to abiotic stress, WRKY18 and WRKY60 have been shown to positively control the response to salt and osmotic stress, as well as ABA sensitivity; this function is counteracted by WRKY40^42^. It has been proposed a complex molecular model where these three WRKY factors may alternatively cooperate or play antagonistic roles to control the expression of the *ABA INSENSITIVE 4* and *5 (ABI4* and *ABI5*) genes^43^. More recently, it has been demonstrated that both WRKY18 and WRKY40 are localised in nuclear bodies (NBs), discrete structures where the photoreceptor phyB, mainly in its active form Pfr^44^, co-localise, and physically interact, with PIF3 and PIF4^24^. Here, we provide evidence that DAG1 negatively controls the expression of both *WRKY18* and *WRKY40*, and that it directly binds the *WRKY18* promoter. Recently, it has been shown that *WRKY6* is repressed during seed germination and early seedling development, and is induced by exogenous ABA and involved in ABA signaling in these developmental processes^45^. We show that *WRKY6* is up-regulated in *dag1* hypocotyls, suggesting that DAG1 represses the expression of *WRKY6* to promote hypocotyl elongation by negatively controlling ABA signaling. Consistently, we show here that the difference between *dag1* and wild type hypocotyl length becomes not significant at higher concentration of ABA, indicating that ABA compensates the lack of DAG1 activity in the *dag1* mutant.

Ethylene has opposite effects on hypocotyl elongation depending on light conditions^19,46^, promoting elongation in the light while suppressing it in the dark^4,47^. In promoting hypocotyl elongation, ethylene functions through PIF proteins positively controlling *PIF3* transcription in the light^6,48^.

Among the genes up-regulated in *dag1* hypocotyls are also seven members of the large *ERF* family encoding transcription factors with very diverse functions including ethylene and ABA signaling^24,25^. Among the *ERF* genes up-regulated in *dag1* hypocotyls, *ERF11* is the best characterised and represses the *ACS2/5* ethylene biosynthetic genes^49^. Interestingly, ABA induces *ERF11* thus repressing ethylene production.

We therefore suggest that DAG1 negatively controls ABA signaling, thus repressing *ERF11* and increasing ethylene biosynthesis, ultimately promoting hypocotyl growth in the light.

Ethylene affects hypocotyl growth via different hormones including auxin, as inhibition of auxin transport, biosynthesis or perception suppresses ethylene-promoted hypocotyl elongation^50^. Consistently, our genome-wide analysis also indicates that four auxin-responsive *SAUR* genes - *SAUR50*, *SAUR63*, *SAUR65* and *SAUR67 -* are down-regulated in *dag1* hypocotyls; three of these, *SAUR50*, *SAUR63* and *SAUR67* are induced by ethylene^50^. In Arabidopsis there are 79 *SAUR* genes, originally identified because of their rapid induction by auxin^51^. More recently, an organ-specific genome-wide analysis characterised 32 *SAUR* genes whose expression is light-repressed in hypocotyls^52^ suggesting a positive role of these genes in hypocotyl elongation. Four of these SAUR genes are the ones down-regulated in *dag1*.

It was also shown that *SAUR50* and *SAUR65* are positively controlled by PIF3 and PIF4, which directly bind their promoters^52^. Consistently, the *SAUR* genes downregulated in *dag1* are among the DE genes in hypocotyls of the *dag1*/WT comparison whose promoters are significantly enriched in the PIF-binding G-box motif.

Among these *SAUR* genes, the promoter of *SAUR67* also contains a multiplicity (26) of DOF binding sites: by ChIP analysis we show here that DAG1 directly binds to this promoter. This suggests a cooperative action of DAG1 with PIF proteins in the regulation of *SAUR67* and in promoting hypocotyl elongation.

It has been shown that PIF4 cooperates with the AUXIN RESPONSE FACTORS (ARFs) ARF6 and ARF8 in promoting hypocotyl elongation^53^. The *ARF6* and *ARF8* transcripts are targets of the *MIR167* family of microRNAs^54^. In Arabidopsis, there are four *MIR167* precursor genes (*MIR167A-D*). Expression profiling of these miRNAs showed that *MIR167D*, which has been proposed to be a pseudogene^54^, is primarily expressed in hypocotyls and cotyledons^55^. We show here that the expression of *MIR167D* is affected by inactivation of *DAG1* in opposite ways in hypocotyls and whole seedlings. This suggest that *MIR167D*, rather than a pseudogene, may be a tissue-specific miRNA involved in hypocotyl elongation.

Finally, we have previously shown that DAG1 is also a key component of the molecular network controlling the seed-to-seedling transition in Arabidopsis^17^. Consistently, quite a high number of DE genes in the hp *dag1*/WT comparison group are related to seed-specific functions; in particular, seven *LEA* genes are up-regulated in *dag1* hypocotyls. LEA proteins were first described to accumulate during plant seed dehydration at late stages of embryogenesis^56^. Indeed, the *LEA* genes that are up-regulated in *dag1* hypocotyls are mainly expressed in seeds^57^, suggesting that DAG1 is required to repress the expression of these genes during seed-to-seedling transition.

In conclusion, the resources resulting from our genome-wide analysis substantiate the role of DAG1 in promoting hypocotyl elongation, and provide clues pointing to a role of this transcription factor in regulating and coupling ABA, ethylene and auxin signaling in this developmental process.

## Methods

### Plant material and growth conditions

All *Arabidopsis thaliana* lines used in this work were grown in a growth chamber at 22 °C with 16/8-h day/night cycles and light intensity of 300 μmol/m^−2^ s^**−**1^ as previously described^15^. *dag1* is the allele described in Papi *et al*.^15^, in Ws-4 ecotype, *dag1DAG1-HA* is the transgenic line described in Gabriele *et al*.^14^. Seeds were surface sterilized and plated on MS agar (halfstrength MS, 0.8% agar, pH 5.7) and stratified at 4 °C for 3 days in the dark.

### Phenotypic analysis

For hypocotyl elongation analysis, all seeds were surface sterilized, sown on MS agar and stratified. After germination was induced by white light for 24 h, plates were incubated for 5 days under continuous red light (40 μmolm^−2^s^−1^). Hypocotyl length was measured every day up to five days, or at the 5th day. For ABA experiments, seeds were sown on MS agar with one layer of filter paper 595 (Schleicher & Schull, Dassel, Germany), and 48 h after stratification, seedlings were transferred to plates containing different ABA concentrations (0, 1, 10, 100 and 150 µM). Hypocotyl length was measured after 5 days. Seedlings were scanned and the hypocotyl length was measured using IMAGEJ software. For cell number measurement, four days-old wild type, *dag1* and *dag1DAG1-HA* seedlings, grown on horizontal plates under continuous red light (40 μmolm^−2^s^−1^), were fixed in a ethanol/acetic acid mixture (6:1). The samples were cleared in 100% ethanol, incubated for 30 minutes at room temperature. Than the 100% ethanol has been removed and replaced with 70% ethanol, and incubated for 30 minutes. After removing ethanol, samples were placed in a chloral hydrate/glycerol/water mixture (8:1:2, g:ml:ml). Seedlings were mounted in chloral hydrate mixture and images of the samples were taken with a Nikon coolpix 990 camera mounted on Zeiss Axioskop 2 plus microscope equipped with DIC optics. For each sample, the number of cells in an epidermal cell file without stomata was counted. All experiments were performed with three biological replicates, each with three technical replicates.

### RNA-seq

Sterilized seeds were sown on MS agar and stratified. After germination was induced by white light for 24 h, plates were incubated for four days under monochromatic continuous red light (40 μmolm^−2^s^−1^). Seedlings were collected and frozen in liquid nitrogen in the dark. For hypocotyls about 1000 seedlings grown in this condition have been dissected, then hypocotyls were frozen in liquid nitrogen in the dark. Three biological replicates for both whole seedlings and hypocotyls were processed. Total RNA was extracted and purified as reported below. Any contaminating genomic DNA was removed using on column DNAse digestion. RNA quality was verified on agarose gel and with Agilent Bioanalyzer 2100 (RNA Integrity Number (RIN) >8).

### RNA-seq data processing and detection of differentially expressed genes

RNA-seq reads were mapped to the *A.thaliana* Tair10 genome assembly using STAR2^58^ with default parameters. The gene and transcript annotation from the Ensembl Plant database (http://plants.ensembl.org) was provided during the alignment step. After filtering for uniquely mapped reads, gene-level read counts were obtained using the HTSeq-count algorithm^59^ and then processed using the edgeR package^60^. For each sample, raw gene counts were first converted into CPM (counts per million) and those having CPM < 1 in at least three samples (the minimum number of samples in a group) were filtered out in order to filter lowly expressed genes. Gene expression levels and fold-changes were estimated after TMM (**T**rimmed-**M**ean of **M** values) normalization^61^. Both common (all genes in all samples) and separate (tag-wise) dispersion parameters were estimated using the Cox-Reid model and integrated into a Negative Binomial generalized linear model (NB-GLM). Statistical significance of differential expression was assessed using a GLM-likelihood ratio test and the ‘Benjamini-Hochberg’ correction for multiple testing. A FDR adjusted p-value of 0.05 was set as threshold to define differentially expressed genes.

### Gene ontology analysis

Functional annotation analysis of DE genes was performed using the Singular Enrichment Analysis tool (SEA) from the AgriGO ontology database (http://bioinfo.cau.edu.cn/agriGO/analysis.php ^62^). AgriGO Enrichment results were further processed using the ReviGO tool^63^ in order to reduce redundant GO terms and prioritize the statistically significant representative terms. ReviGo analysis was performed using default parameters: allowed similarity: Medium (0.7); database: whole UniProt; semantic similarity; SimRel. A Fisher’s exact test q-value of 0.05 was used as a threshold for significant enrichment.

### Expression analysis

For RNA extraction, four days-old *dag1* and wild type seedlings, grown under monochromatic red light, were harvested and immediately frozen in liquid nitrogen. Total RNA was isolated by grinding the tissues in liquid nitrogen. The samples were then vortexed for 3 min in the presence of an extraction buffer (0.1 MLiCl, 0.1 M Tris-HCl [pH 8], 0.01 M EDTA, 1% sodium dodecyl sulfate-phenol-chloroform mixture (1:1:1). Three phenol-chloroform extractions were then performed. RNA was precipitated overnight at 4 °C with 1 volume of 4 M LiCl, followed by a second precipitation with 0.1 volume of sodium acetate, pH 5.2. RT-qPCR assays were performed with SYRgreen I master using the LightCycler® 480 instrument (Roche, http://www.roche.com). A total of 1 μl of the diluted cDNA was used, along with the specific primers, listed in Table S3 (Supplementary Table S3). Relative expression levels were normalized with *UBQ10* (At4g05320) reference gene.

### Chromatin Immunoprecipitation (ChIP) assay

ChIP assay was performed with 5 days old-seedlings of the transgenic line overexpressing the DAG1-HA chimeric protein in a *dag1* mutant background and with the *dag1* mutant as a negative control. Seedlings (about 1gr) were washed with water, then resuspended with 3 ml extraction buffer 1 (0.4 M sucrose, 0.01 M Tris-HCl [pH 8], 5 mM β-mercaptoethanol, 1 mM PMSF, 1x protease inhibitors) and treated with 37% formaldehyde for 10 min under vacuum. The reaction was stopped with glycine 0.125 M. Samples were then harvested with a miracloth membrane and immediately frozen and ground in liquid nitrogen. Extraction buffer was added to the samples (30 ml) then filtered on a miracloth membrane. After a centrifugation (4000 g, 20 min), the pellet was resuspended in 1 ml extraction buffer 2 (0.25 M sucrose, 0.01 M Tris-HCl [pH 8], 10 mM MgCl_2_, 1%Triton x-100, 5 mM β-mercaptoethanol, 1 mM PMSF, 1x protease inhibitors). After 10 min on ice, samples were centrifuged (12000 g, 10 min, 4 °C). The pellet was resuspended in 0.3 ml extraction buffer 3 (1.7 M sucrose, 0.01 M Tris-HCl [pH 8], 2 mM MgCl_2_, 0.15%Triton x-100, 5 mM β-mercaptoethanol, 1 mM PMSF, 1x protease inhibitors), then samples were centrifuged again (1 h, 16000 g, 4 °C). The chromatin pellet was resuspended in 0.3 ml lysis buffer (0.05 M Tris-HCl [pH 8], 0.01 MEDTA, 1% SDS, 1 mM PMSF, 1x protease inhibitors). Chromatin was sheared by sonication. To an aliquot of each sample (0.1 ml) was added 0.9 ml ChIP buffer (1.1% Triton, 1.2 mM EDTA, 16.7 mM Tris-HCl [pH 8], 167 mM NaCl, 1 mM PMSF, 1x protease inhibitors).

The immunoprecipitation was performed using HA-probe antibody (Y-11, sc-805 Santa Cruz), or without antibodies as negative control, overnight at 4 °C. After reverse cross-linking, the enriched DNA levels were quantified by qPCR using specific primer sets (Supplementary Table S3). The Fold enrichment of a specific region was calculated respect to the negative control without antibody. The values are the average of two biological replicates presented with SD values. Significant fold enrichments were analyzed by *t*-test (*P ≤ 0,05).

### Statistical analysis

Two-way ANOVA followed by Tukey’s HSD posthoc test were used for pairwise multiple comparison (Figs 1 and 7). For hypocotyl elongation assays, statistical significance is indicated by the use of different letters. The values of gene expression analysis and ChIP assays are the mean of three biological replicates presented with SD values. Significant differences were analyzed by t-test (*P ≤ 0,05; **P ≤ 0,01).

## Electronic supplementary material

Supplementary Information

Additional File 1

Additional File 2

Additional File 3
