# Supplementary material for: Genome-wide RNA-seq analysis indicates that the DAG1 transcription factor promotes hypocotyl elongation acting on ABA, ethylene and auxin signaling
Source: Sci Rep. 2018 Oct 26;8:15895. doi: 10.1038/s41598-018-34256-3 (PMC6203721; doi:10.1038/s41598-018-34256-3)
Supplement: Supplementary file 1 — Supplementary Information [file 41598_2018_34256_MOESM1_ESM.pdf]

Genome-wide RNA-seq analysis indicates that the DAG1 transcription factor promotes hypocotyl elongation acting on ABA, ethylene and auxin signalling

Lorrai Riccardo[1], Gandolfi Francesco[2], Boccaccini Alessandra[1], Ruta Veronica[1], Possenti Marco[3], Tramontano Anna[2], Costantino Paolo[1], Lepore Rosalba[2]*, Vittorioso Paola[1]*

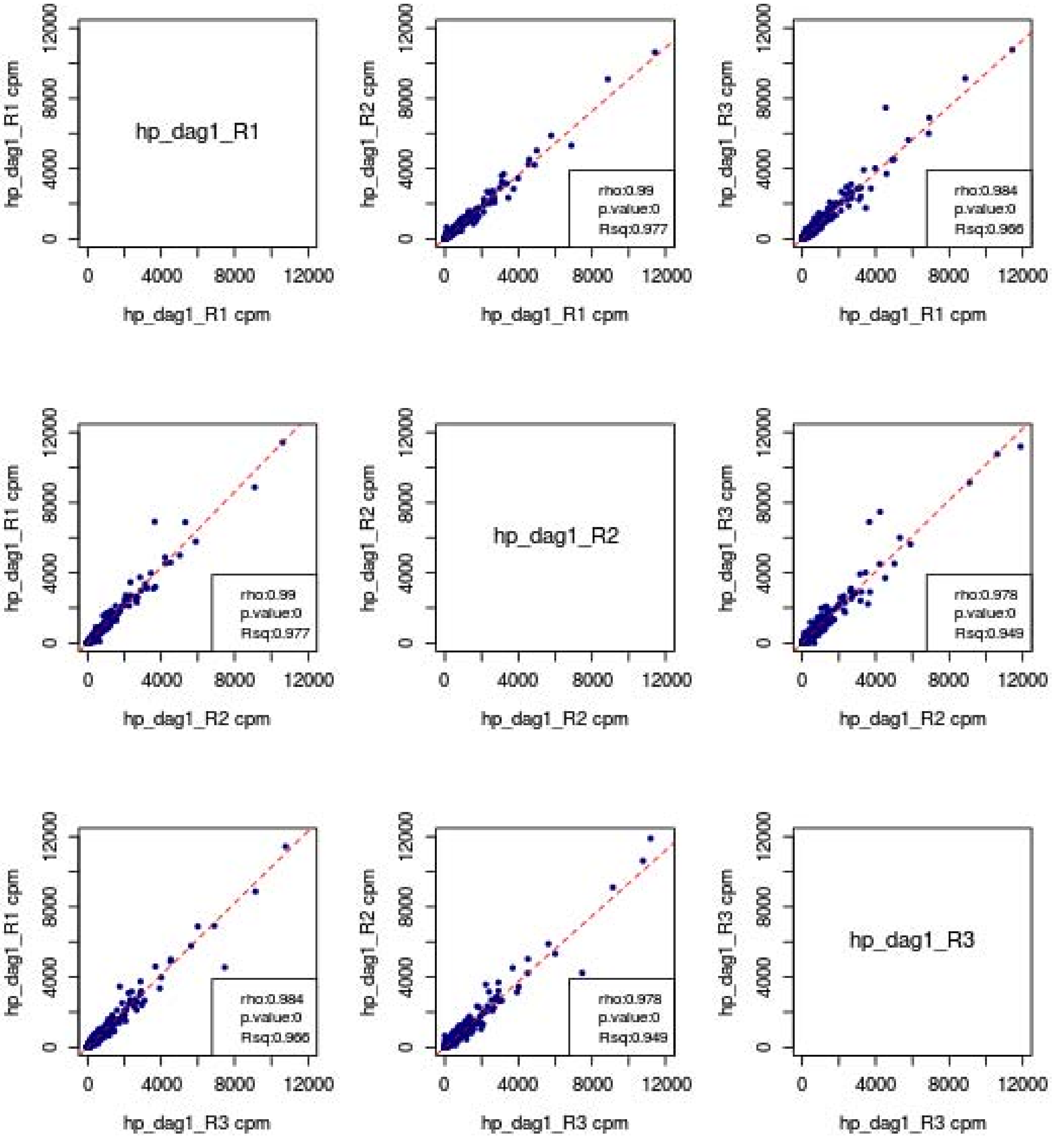

Supplementary Fig S1. Scatter plots of CPM values between replicates and Spearman's correlation coefficients.

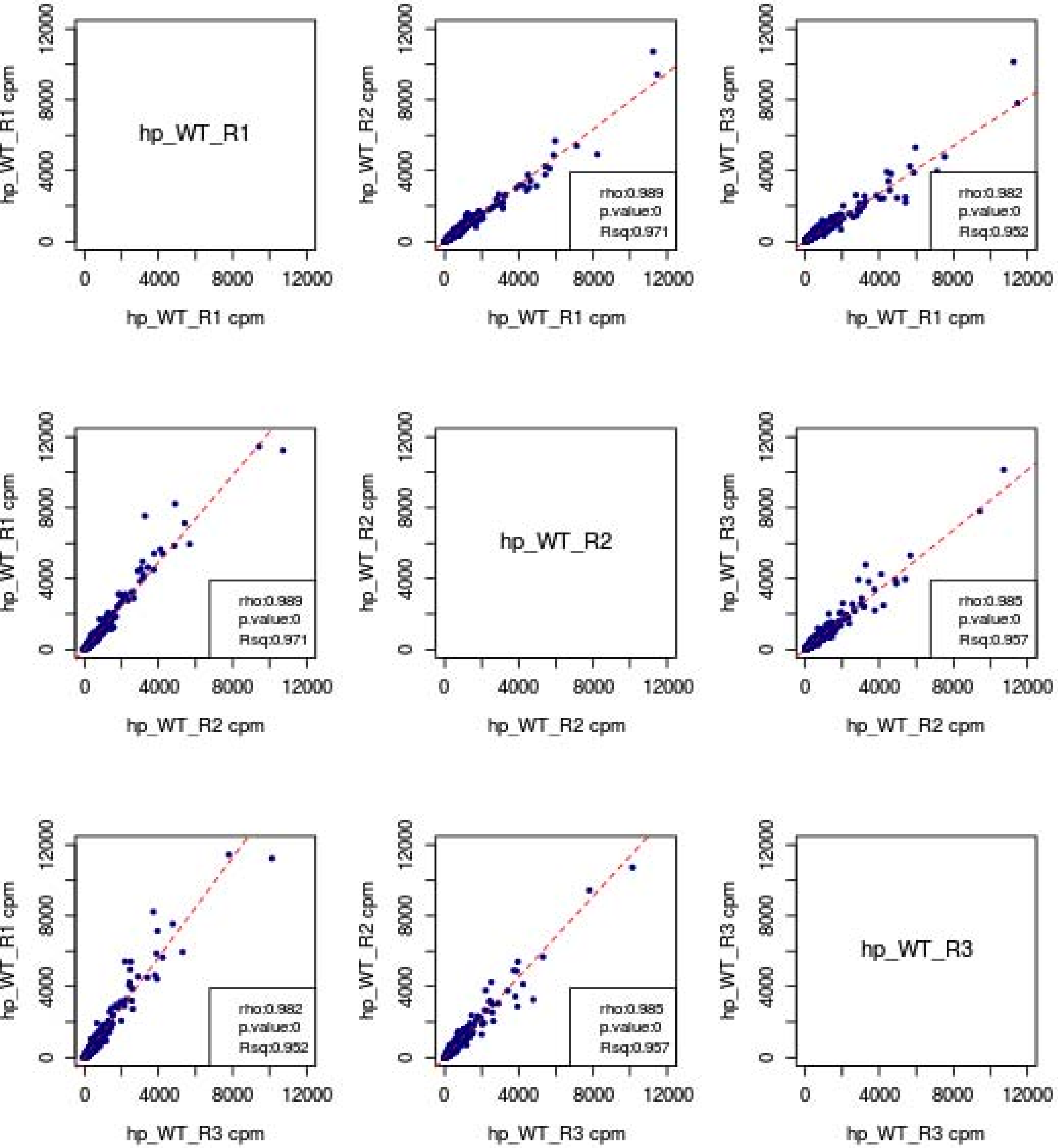

Supplementary Fig S2. Scatter plots of CPM values between replicates and Spearman's correlation coefficients.

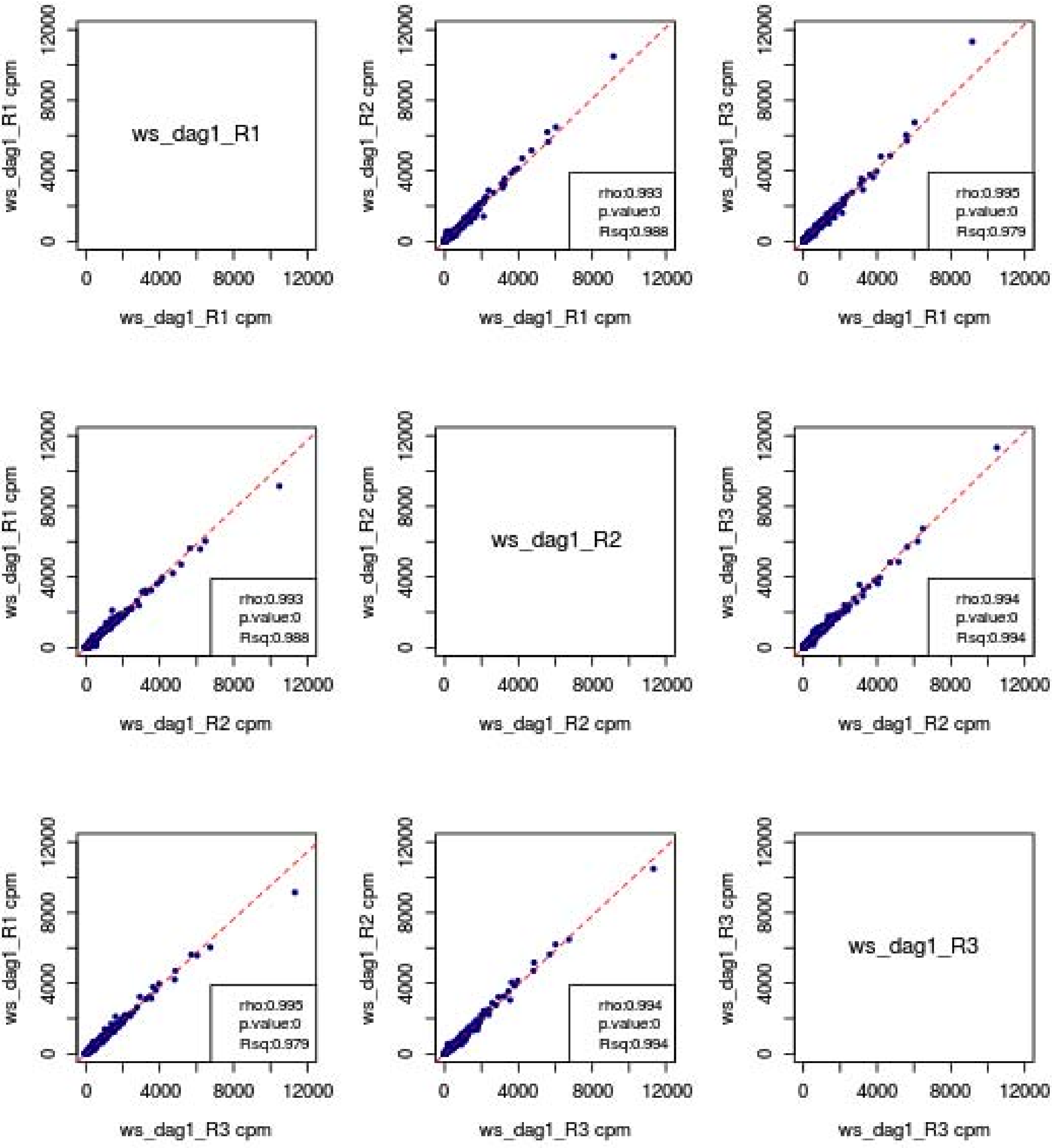

Supplementary Fig S3. Scatter plots of CPM values between replicates and Spearman's correlation coefficients.

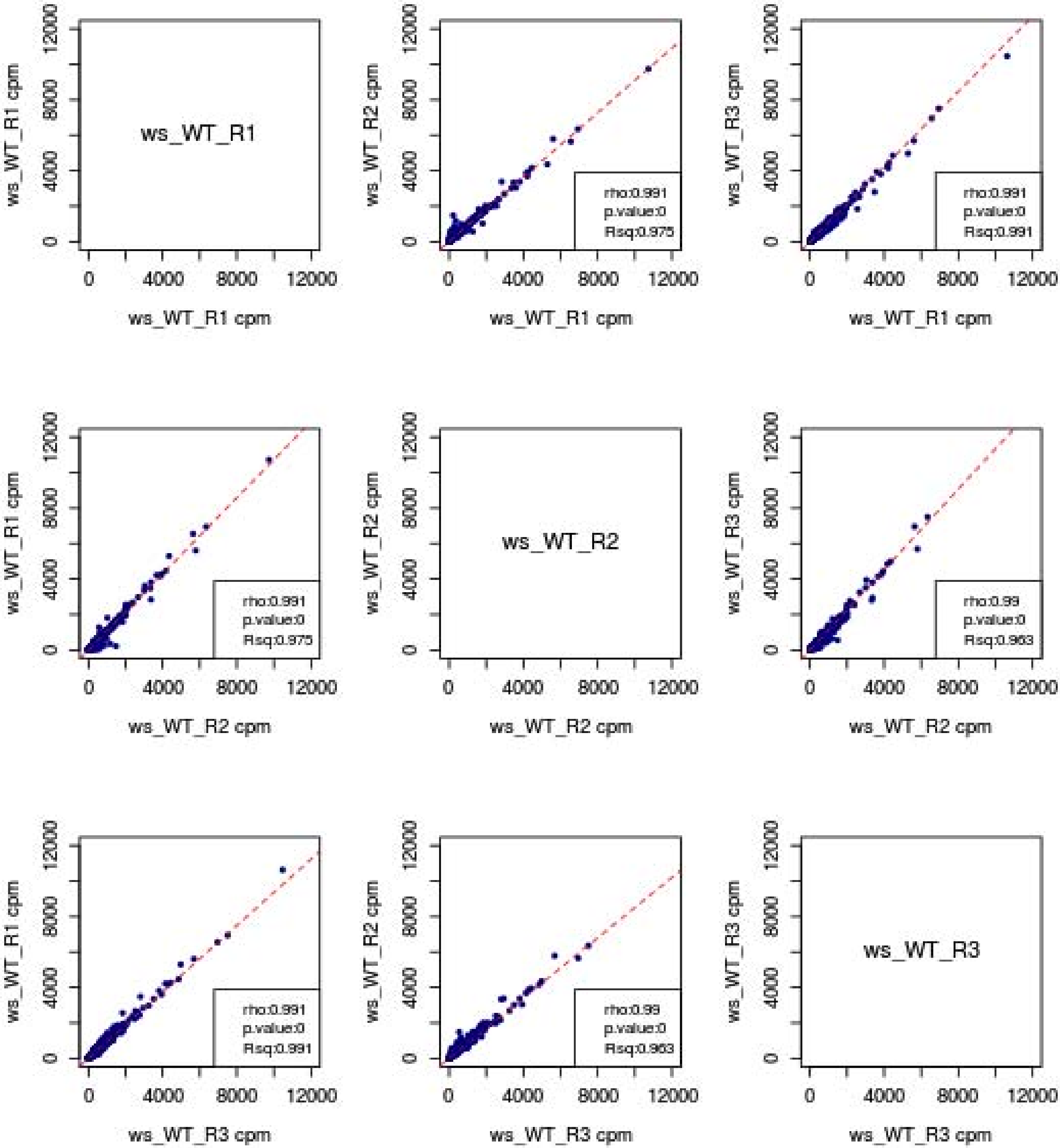

Supplementary Fig S4. Scatter plots of CPM values between replicates and Spearman's correlation coefficients.

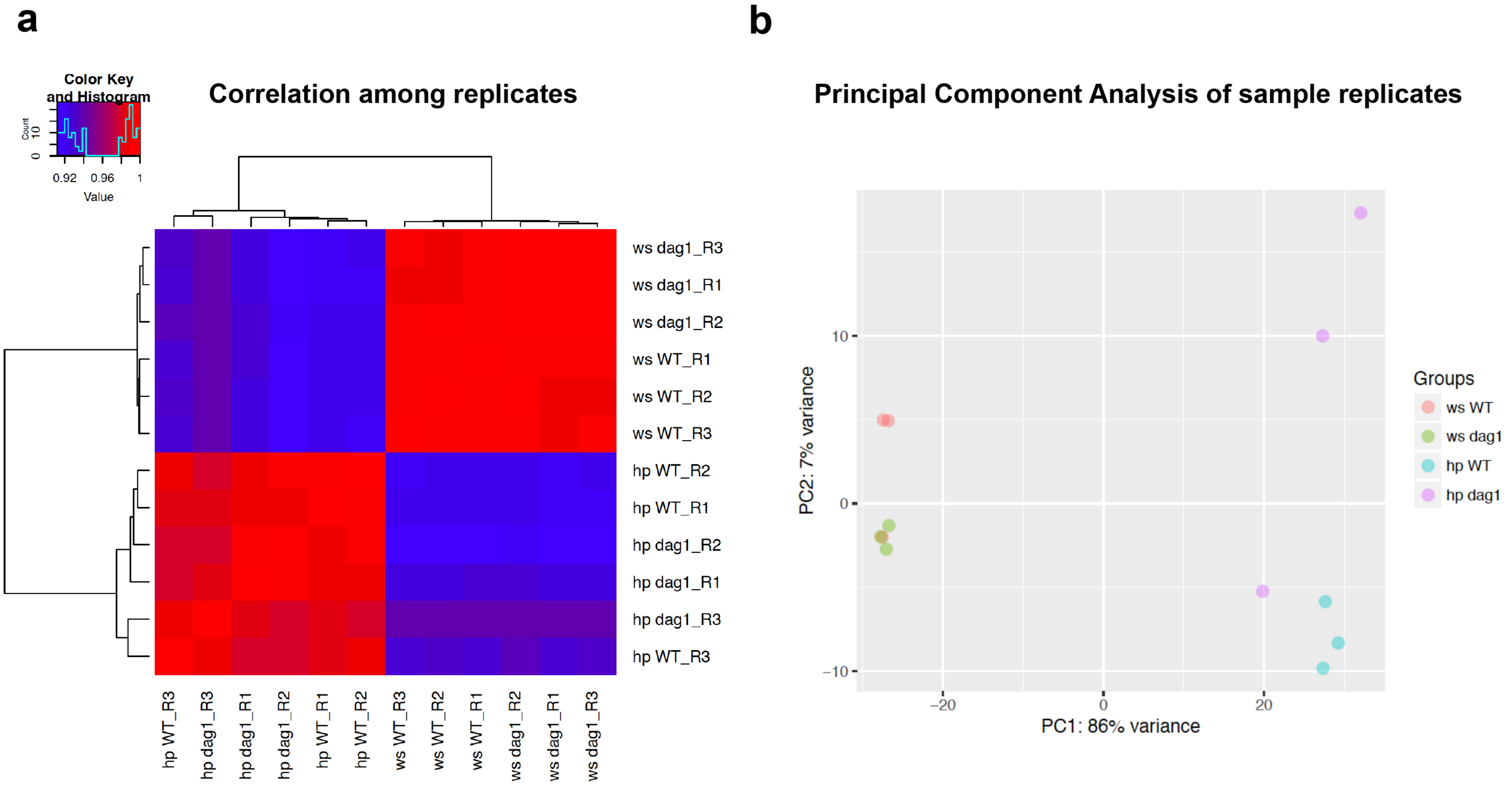

Supplementary Fig S5. **Sample clustering and Principal Component Analysis of RNA-seq data. (a)** Correlation clustering of RNA-seq replicates according to normalized gene expression values. Spearman correlation was used a distance metric (defined as 1-Spearman's correlation coefficient) and the "average" method as linkage function. **(b)** Principal Component Analysis (PCA) was performed using the 'plotPCA' function provided within the 'DESeq2' (v1.18) Bioconductor R package.

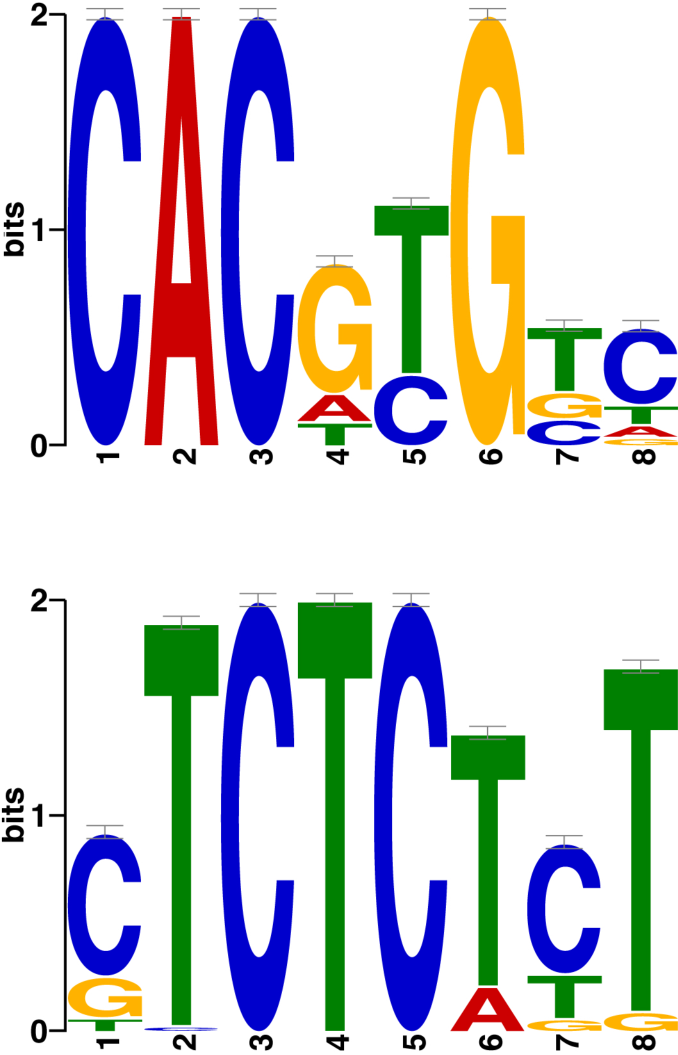

Supplementary Fig S6. The PIF binding motif is enriched in DE genes. Hidden Markov model sequence logos obtained using MEME[1]/TOMTOM[2] for significantly enriched motifs found in DE genes promoter sequences.

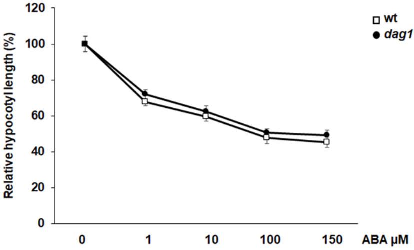

Supplementary Fig S7. Relative hypocotyl length of *dag1* (black) and wild type (white bar) seedlings grown under red light for five days, with increasing concentration of ABA (0, 1, 10 100 and 150 µM). The values, normalised to mock treated samples for both genotypes, are the mean of three biological replicates, presented with SD values.

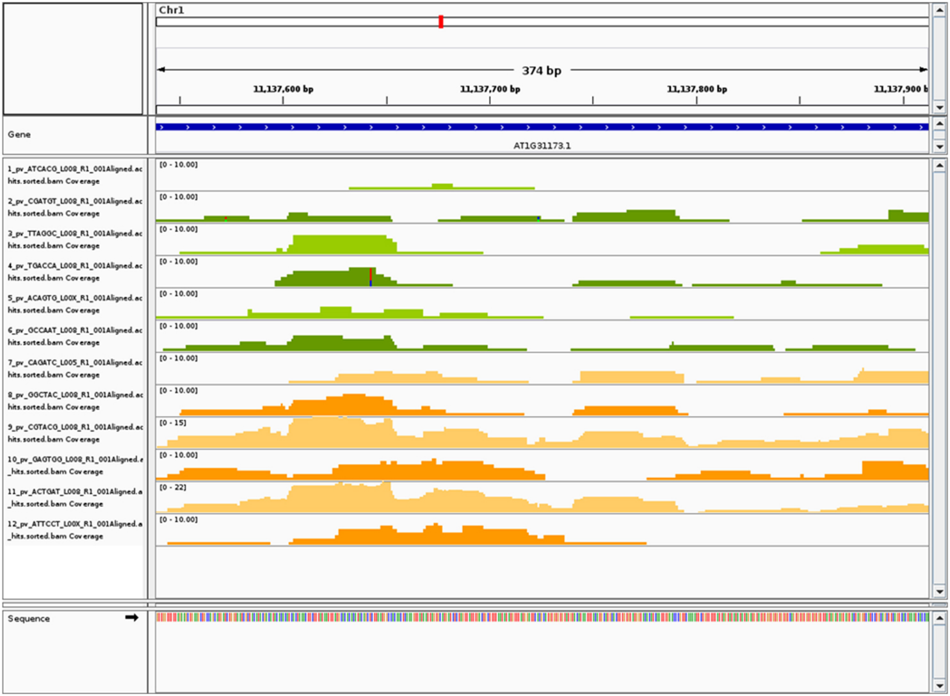

Supplementary Fig S8. Relative sequence read abundance of *miRNA167D* (Integrative Genome Browser view) as histograms, in *dag1* and wild type hypocotyls and seedlings. The Y-axis indicates read depth with the same scale for all samples (three biological replicates). The green profiles are referred to the whole seedlings, whereas the orange profiles are referred to the hypocotyls (*dag1* in dark green/orange, wild type in light green/orange). The miRNA167 locus is shown by the blue track in the top panel.

Supplementary Table S1. Reads mapped to the *Arabidopsis thaliana* genome (TAIR10)

Sample	Tot Reads	% Uniquely mapped	% Multi Mapped	% Unmapped
WT 1 (ws)	18967605	92	7,25	0,75
dag1 1 (ws)	27654782	91	7,9	1,08
WT 1.2 (ws)	25388619	91,25	7,88	0,87
dag1 1.2 (ws)	29184780	91,06	7,74	1,2
WT 1.3 (ws)	21427524	91,98	6,96	1,05
dag1 1.3 (ws)	24563037	91,92	6,84	1,24
WT 1 (hp)	19885967	90,04	5,82	4,05
dag1 1 (hp)	19617630	92,35	6,24	1,4
WT 1.2 (hp)	31727863	93,48	5,42	1,1
dag1 1.2 (hp)	31503405	93,22	5,43	1,36
WT 1.3 (hp)	24355880	92,7	6,52	0,78
dag1 1.3 (hp)	20956766	92,72	5,8	1,47

Supplementary Table ~~S3~~. List of the seed-specific genes differentially expressed in *dag1* hypocotyls respect to wild-type.

**Name**	**Locus**	**Function**
EM6	AT2g40170	LEA
LEA18	AT2g35300	LEA
M10	AT2g41280	LEA
M17	AT2g41260	LEA
	AT2g23110	LEA
	AT3g17520	LEA
	AT3g53040	LEA
CRA1	AT5g44120	seed storage proteins
PAP85	AT3g22640	seed storage proteins
SESA5	AT5g54740	seed storage proteins

Supplemental Table 3.

Specific primer sets used in the qRT-PCR:

	Forward	Reverse
UBQ10	GGCCTTGTATAATCCTGATGAATAAG	GGCCTTGTATAATCCTGATGAATAAG
RAB18	CGATGGGCGGCATGGGAT	CACCACCGGGAAGCTTTTCCTT
ABR1	TCCGTTGCGTTTCGGTCACA	CTGATTCTGGTGGCGGCTGAA
AT2G23110	ACCGATGCTCCCACTCCTTCA	CGGAACGCCCTGACGGTT
LEA18	CTAAGGAGGCGCAGGCCAAA	CTCGGGTAGGGTAGGTGGAGTG
EM6	GGAGCCGAGGAGGGCAAAC	CTCAGCGTGTTCCCCACCAG
M17	TGGTGGTTGTAGGTGGGGATGTT	CAACAGTTTCCACAACCTCGTTTGC
M10	TGGTGGTGGTTGTAGGTTCGGA	CTTCGTTGGCCTCGGCTTGA
AT3G17520	CGAAAGATAAGGCGTCGCAGAGTT	GCTCAGCATCATCGTCGGTCAA
AT3G53040	GCCCATGGAGACGAGAGCAC	CCGCCTCTTTCAGTACCGTCG
CRA1	GGTGGTGAAACGCGCAACAA	GACTGAGGTTCGTCCCGC
WRKY6	TCTCGGGGCTGCAGTTCTCT	GGTCAGCTGTGAGTGCCGTT
WRKY18	GGGATGCTACAAGCCAGGGTG	TCCCTTTCGTTTTTCTCCAACGCT
WRKY28	GCAGACCTCATGACTCCAAGAAGC	ATAATCCAAAGCCGCCGCCA
WRKY33	ACCATCGGTTGTCCAGTGAGGA	AACCGCTACCACGAGCTGC
WRKY40	GCTTGACTGTGCCGGTGACT	GGAAGAAGCCATTTGCTCCACCA
WRKY46	CTGCACCTGCTGCTGTTGAGAA	CGACCACAACCAATCCTGTCCG
WRKY70	CAGGCCAGTTACGTCAATGGGAAAA	GAAATCGCCGCCACCTCCA
SAUR50	GAGATGGTTAAAGAAGAAGCGG	CCTAGATGGGCTTAAAAATAAAGG
SAUR63	TGTAGTACTACGCAACAGTTGC	GAAATGATCTGTCTATCTAATCCAC
SAUR65	CCACAAGAACAACAGAGTGGTT	ACAGAGGAAAAAATGTCTACACTC
SAUR67	CAGAAAAGGCTCTGTTGATGTC	GTTGAGTACTCTGTTCTTGCTG
ERF2	AATTCCGGTGAACCTGACCC	ACGACGTAGAAGAGGACGAC
ERF5	CCGATGAAGGTGAGAAGAAAC	GTCAACGCTCTGTTCCGTCT
ERF11	TAAGGCTGGGATGATGGTGT	AAAACCACACGTCGTCCTTC
ERF105	CCGGTGGTTCAAGAGAATGAT	CCTAACCAGACACGAACACC
ERF109	CAGTTGAAGCAGAGCAATGG	CCATTCCCAAAATCCATCATC

Specific primer sets used for chIP:

	Forward	Reverse
WRKY18 c	CAGCTTTCTATGTCTTGTTGTGC	AAAGCTGTCTTCTATAAAGTCCAG
WRKY18 b	CTGGACTTTATAGAAGACAGC	AGATAGGTTTTGGGATATGTTAGG
WRKY18 a	CGACAGTAAATTTAACCCATGCAT	ACACTGATTGGCTAAAAATATGGG
ERF2 c	CCCACTAAACTAAAACAAATTTGG	ATGTGGAAGGTGGTTAAGAGG
ERF2 b	GACATTGGCAATTAGGAAAATTCT	ATAATTATATGAATGGGGACACTG
ERF2 a	CCAAATCATTGGTGTACAAAAAG	GGAGTAGTGAAATCATAGTGATA
SAUR67 c	CTCTTTCTTTCTCCACACTTTGC	CTTCATAAGCTTCTTTGCGTTTATC
SAUR67 b	CAACCACAACTTTCATTCAAAGAG	GGAGAGTTAAAGCTTGAGATTGG
SAUR67 a	TTTACCTGATGGACTACTAACCAA	AAGCCAAAAATACCAAACTCATGC

References

1 Bailey, T. L. & Elkan, C. Fitting a mixture model by expectation maximization to discover motifs in biopolymers. *Proc Int Conf Intell Syst Mol Biol* **2**, 28-36 (1994).

2 Gupta, S., Stamatoyannopoulos, J. A., Bailey, T. L. & Noble, W. S. Quantifying similarity between motifs. *Genome Biol* **8**, R24, doi:10.1186/gb-2007-8-2-r24 (2007).
